# Developing an electronic portfolio of learning for family medicine training in South Africa

**DOI:** 10.4102/phcfm.v16i1.4525

**Published:** 2024-06-28

**Authors:** Louis Jenkins, Robert Mash, Mergan Naidoo, Ts’epo Motsohi

**Affiliations:** 1 Department of Family and Emergency Medicine, Faculty of Medicine and Health Sciences, Stellenbosch University, Cape Town, South Africa; 2 Primary Health Care Directorate, Department of Family, Community and Emergency Care, Faculty of Medicine and Health Sciences, University of Cape Town, Cape Town, South Africa; 3 Department of Family and Emergency Medicine, George Hospital, Western Cape Department of Health, George, South Africa; 4 Discipline of Family Medicine, School of Nursing and Public Health, College of Health Sciences, University of KwaZulu-Natal, Durban, South Africa

**Keywords:** electronic, portfolio of learning, postgraduate, training, assessment, family medicine, South Africa

## Abstract

Workplace-based assessment has become increasingly crucial in the postgraduate training of specialists in South Africa, particularly for family physicians. The development of a Portfolio of Learning (PoL) has been a central focus within the discipline of family medicine for over a decade. Initially, a paper-based portfolio was adopted to collect evidence of learning for 50 out of 85 agreed exit-level outcomes. Stellenbosch University led the conversion of this portfolio into an electronic format, known as e-PoL, utilising Scorion software. The e-PoL was successfully implemented in the Western and Eastern Cape regions and was subsequently adopted nationally under the coordination of the South African Academy of Family Physicians. In 2023, the e-PoL underwent a redesign to gather evidence of learning for 22 entrustable professional activities (EPAs). Key insights from this development process underscore the importance of the PoL in supporting assessment-for-learning rather than merely assessment-of-learning. This necessitates features for feedback and interaction, ensuring that the PoL functions beyond a mere repository of forms. Additionally, the e-PoL should facilitate triangulation, aggregation, and saturation of data points to effectively measure EPAs. Furthermore, the PoL has not only documented learning but has also played a pivotal role in guiding the development of clinical training by explicitly outlining expectations for both registrars and supervisors. While the initial design and development costs are significant, operational costs become affordable when shared across all training programmes.

## Introduction

Postgraduate family medicine training in South Africa occurs in health services. Workplace-based assessment (WPBA) is becoming more critical for high-stakes decision-making.^1^ Each of the nine universities is embracing WPBA to make decisions on progression through the 4-year training programmes, and the College of Family Physicians is strengthening the role of WPBA in the national fellowship examination.

Workplace-based assessment requires four components.^2^ Firstly, the goals of WPBA are defined in terms of entrustable professional activities (EPAs). The development of EPAs has been described in a separate short report.^3^ Secondly, WPBA requires effective clinical training by skilful supervisors within a supportive learning environment. The development of clinical trainers has also been described in another short report.^4^ Thirdly, the evidence of learning and competence is collected in a portfolio of learning (PoL) that provides the data for WPBA. Lastly, the evidence collected in the PoL is scrutinised and assessed by a competency committee, which makes a summative decision. This short report elaborates on developing the electronic PoL for postgraduate family medicine training in South Africa.

## Design of the paper-based portfolio of learning

In 2010, the discipline of family medicine in South Africa agreed on 85 learning outcomes for postgraduate training aligned to five unit standards.^5^ These unit standards focus on leadership and governance, clinical work, family- and community-orientated primary care, clinical training and capacity building, ethics and professionalism. Summative decisions on competence were made using traditional methods, which included oral examinations, written papers, objective structured clinical examinations and simulated consultations. These methods were removed from the workplace, could not assess all the required competencies and sometimes lacked validity.

At the same time, the assessment of specialists at the end of training became the responsibility of the Colleges of Medicine of South Africa (CMSA). They required every candidate to present an acceptable PoL to enter the licensing examination. The discipline of family medicine participated in a national Delphi study and reached a consensus that 50 of the 85 learning outcomes should be assessed by the PoL.^6^ The same Delphi study recommended several assessment methods based on fundamental principles. The portfolio was designed and implemented nationally and evaluated in terms of its acceptability, educational impact, usefulness for assessment, the experience of registrars and supervisors and reliability.^7,8,9^ The final portfolio was paper based and implemented by all training programmes. The contents of the PoL are listed in Table 1.

**TABLE 1:** Contents of the national paper-based portfolio of learning.

Number	Element in the portfolio	Description
1	Introduction to your portfolio	Overview of the contents and guidance on how to use the portfolio
2	Learning outcomes	Explanation of how the portfolio related to the 85 learning outcomes and unit standards
3	Learning plans, reflections on allocations and supervisor’s assessments	A minimum of two learning plans, reflections and assessments per year were required. Allocations could be within the generalist setting (e.g. wards in a district hospital) or to specialist settings (e.g. disciplines in a regional or tertiary hospital)
4	Educational meetings with the supervisor	Registrars needed to meet regularly with their supervisor for educational interactions
5	Observations of the registrar by the supervisor	A minimum of 20 observations were required per year of consultations, procedures and at least one teaching event.
6	Written assignments	Registrars could include written assignments from their academic programmes, which demonstrated their application of theory in the workplace, for example quality improvement projects, community diagnosis.
7	Logbook of clinical skills	The 214 clinical skills were categorised into a number of logbooks that needed to be completed twice a year by the registrar and validated by the supervisor. These assessed their competency on a 4-point scale.
8	Emergency medicine certificate(s)	Certificates from emergency medicine courses such as advanced trauma and life support, advanced paediatric life support or advanced cardiac life support.
9	Other courses, workshops, conferences	Other evidence of learning from courses, workshops or conferences
10	End-of-year assessment	A portfolio assessment tool collected data on the completeness of the portfolio and adherence to the requirements. The Head of Department made a final assessment on the quality of the portfolio.

## Conversion to an electronic portfolio

Stellenbosch University (SU) explored the possibility of developing an electronic version to replace the paper-based PoL. A key issue was the cost needed to design and develop a PoL while ongoing operational costs were affordable in South Africa. Some training programmes attempted to use existing course management software (e.g. Moodle) or research software (e.g. REDCap). However, these could not provide the required educational functionality, such as trainee reflections and supervisor feedback from observed clinical practice.

Many postgraduate training programmes in other regions are using e-PoLs.^10^ After an exploration of the software options available, SU identified EPASS (Electronic Portfolio and Assessment Support) as a product that would support our content and educational principles.^11^ The EPASS portfolio was developed, partly funded by a research grant and implemented. However, the EPASS company withdrew from South Africa, necessitating the search for an alternative. Without a locally developed version, we identified Scorion, an online portfolio for programmatic assessment that digitally records and provides feedback on educational activities in the form of data.^12^ The developers fortunately negotiated a grant from the Dutch government to support the development of an e-PoL for Family Medicine in South Africa.

An evaluation compared the e-PoL to the previous paper-based version.^13^ The researchers concluded that ‘overall the e-portfolio was an improvement on the paper-based portfolio because it was more accessible, user-friendly, secure, structured, enabled better monitoring of progress and improved the quality of feedback’.^13^ Although the e-PoL requires an internet connection, the EPASS and Scorion portfolios were also successfully implemented at Walter Sisulu University, which works in one of the most rural and impoverished provinces of South Africa.

Although SU developed Scorion, it remained aligned with the nationally agreed PoL content and structure. This allowed SU to offer the Scorion e-PoL to all family medicine training programmes in the country and for the SA Academy of Family Physicians to coordinate the management platform at a national level. Eight of the nine training programmes adopted the Scorion e-PoL and shared the costs, with the previously disadvantaged universities accommodated in the cost sharing. The redesign of the Scorion portfolio cost approximately R400 000. The ongoing operational costs include an annual licence fee (R12 500/institution) and an annual fee per supervisor (R60) and registrar (R650) to register on the system. There is also a fee for the service desk that is determined by the number of hours of support required from Scorion. This is anticipated to cost R70 000 over 2 years.

## Revision of the electronic portfolio for entrustable professional activities

After the e-PoL was implemented across the country, the CMSA and the National Committee of Medical Deans agreed on an initiative to strengthen WPBA. The discipline of family medicine reached a national consensus on 22 EPAs, which necessitated a redesign of the e-PoL.^3^ Although the portfolio’s content remained largely the same, the redesign process provided an opportunity for a focused review of content with updated adjustments to required skills. The process also required aggregating the ‘data points’ represented by each portfolio entry to measure the 22 EPAs. This meant that each ‘form’ was redesigned to link it to the relevant EPAs, and new reports were created to display the quantitative and qualitative evidence for each EPA. Wherever possible, the clinical supervisor had to record an ad hoc entrustment decision for the registrar for that data point on a scale from 1 to 5, as follows:

1. Can observe only.
2. Direct supervision (the supervisor must be next to the registrar).
3. Indirect supervision (the supervisor must be available in the facility).
4. Distant supervision (the supervisor can be available off-site at a distance).
5. Supervising others (no supervision is needed).

The process of annual portfolio assessments was replaced by a local clinical competency committee that would make a summative entrustment decision for each EPA twice a year based on the evidence provided.^14^ The design of the new version of the e-PoL is summarised in Table 2.

**TABLE 2:** Content of the electronic portfolio of learning redesigned for workplace-based assessment.

Number	Element in the portfolio	Description
1	My shared folders	A collection of 21 documents that introduce the portfolio, provide a preamble to the EPAs and the 22 EPAs and provide background reading on WPBA.
2	Learning plans, reflections on allocations and supervisor’s assessments	A minimum of two learning plans, reflections and assessments per year are required. Allocations can be within the generalist setting (e.g. wards in a district hospital) or to specialist settings (e.g. disciplines in a regional or tertiary hospital)
3	Educational meetings with the supervisor	Registrars need to meet regularly with their supervisors for educational interactions. Tools are available to assist: Significant event analysis tool Entrustment based discussions
4	Observations of the registrar by the supervisor	As many observations as possible. A variety of tools are available: Mini-Clinical Evaluation Exercise (Mini-CEX) (for the consultation) Communication skills observation tool Direct observation of procedural skills (DOPS) (for procedures) Caesarean section observation tool Anaesthetic observation tool Teaching and/or presentation assessment tool (for group teaching events) One-minute preceptor tool (for one-on-one teaching)
5	Multisource feedback	Multisource feedback on performance and entrustability once a year from 10 to 20 people who report to you, work alongside you or supervise you.
6	Written assignments	Registrars can include written assignments from their academic programmes that demonstrate their application of theory in the workplace, for example, quality improvement projects and community-oriented primary care projects. The assessment of the assignments is aligned to the entrustment scale.
7	Logbook of clinical skills	The 245 clinical skills are categorised in a mutually exclusive approach into the 22 EPAs. The logbooks record exposure to or experience with each skill over the training programme.
8	Emergency medicine certificate(s)	Certificates from emergency medicine courses such as advanced trauma and life support, advanced paediatric life support or advanced cardiac life support.
9	Other courses, workshops, conferences	Other evidence of pertinent learning from courses, workshops or conferences
10	Clinical Competency Committee form	An interim and final form that allows the competency committee to record a summative entrustment decision for each EPA based on the quantitative and qualitative data in the registrar PoL report generated by Scorion.

EPA, entrustable professional activity; WPBA, workplace-based assessment.

## Key lessons learnt

The PoL is more than simply a repository for forms that assess learning but should support the educational principle of ‘assessment-for-learning’.^15^ Each data point in the PoL should be an opportunity for constructive feedback or reflection that stimulates further learning. Therefore, clinical trainers must be skilful in providing such feedback and recording it in the PoL. Faculty development is needed to enable this. Too often, clinical trainers simply record scores with no feedback, fail to guide future learning or provide generic and vague feedback that cannot easily be implemented. In our PoL, we have emphasised feedback structured into three questions: what was done well, what could be done better and what the learner needs to do next (an action plan). In addition, we have moved away from scores in the various forms to ad-hoc entrustment decisions. This approach to the design and use of the e-PoL emphasise the importance of trust between registrars and trainers, the relevance of the clinical context and the creation of a learning culture that facilitates WPBA.^16,17^

The e-PoL should support key principles of WPBA, namely triangulation, aggregation and saturation. Triangulation implies that there are various data points from different assessors in different settings to support an entrustment decision on each EPA. Aggregation requires that the data points be designed in such a way that they can be aggregated to measure the EPAs. Saturation is achieved when enough data points support an entrustment decision on the EPA. In this way, the PoL also supports ‘assessment-of-learning’. These data points then serve as evidence in making summative entrustment decisions for each EPA to ascertain readiness for the national exit exams by the end of the third year of study.

In South Africa, the speciality of family medicine was established in 2008, and the PoL has been developed alongside the training programmes. In this context, the PoL also acted as a guide and a benchmark for what was expected from clinical trainers and registrars in the workplace. It did not just document what happened but shaped the clinical training itself and made expectations explicit. The e-PoL allowed programme coordinators to monitor the performance of both registrars and clinical trainers continuously. Regular feedback helped ensure that registrars completed the PoL over the whole year and that supervisors were aware of their contributions relative to their peers. The migration to the e-PoL (and the subsequent development of EPAs) also stimulated other engagements and reflections on the curriculum content, allowing for focused updates and changes in the range of required skills.

In contrast to the paper-based portfolio, the e-PoL made summative assessment by the clinical competency committee much easier as data could be automatically aggregated and displayed in reports, graphs or radar diagrams (dashboard).

## Conclusion

Developing an e-PoL for postgraduate family medicine training in South Africa has been a complex process to clarify what clinical training and supervision are needed, source funding and develop a feasible and valuable digital platform. It has also involved getting people to engage with it early on and supporting users with a management platform. Lessons learnt could be helpful for other family medicine programmes and clinical disciplines in similar contexts in sub-Saharan Africa.
